# Sirtuins: Key players in obesity-associated adipose tissue remodeling

**DOI:** 10.3389/fimmu.2022.1068986

**Published:** 2022-11-24

**Authors:** Jiali Chen, Ruohan Lou, Fei Zhou, Dan Li, Cheng Peng, Ligen Lin

**Affiliations:** ^1^ State Key Laboratory of Quality Research in Chinese Medicine, Institute of Chinese Medical Sciences, University of Macau, Taipa, Macao SAR, China; ^2^ State Key Laboratory of Southwestern Chinese Medicine Resources, School of Pharmacy, Chengdu University of Traditional Chinese Medicine, Chengdu, China; ^3^ Department of Pharmaceutical Sciences and Technology, Faculty of Health Sciences, University of Macau, Taipa, Macao SAR, China

**Keywords:** Sirtuins, obesity, adipose tissue remodeling, inflammation, fibrosis

## Abstract

Obesity, a complex disease involving an excessive amount of body fat and a major threat to public health all over the world, is the determining factor of the onset and development of metabolic disorders, including type 2 diabetes, cardiovascular diseases, and non-alcoholic fatty liver disease. Long-term overnutrition results in excessive expansion and dysfunction of adipose tissue, inflammatory responses and over-accumulation of extracellular matrix in adipose tissue, and ectopic lipid deposit in other organs, termed adipose tissue remodeling. The mammalian Sirtuins (SIRT1–7) are a family of conserved NAD^+^-dependent protein deacetylases. Mounting evidence has disclosed that Sirtuins and their prominent substrates participate in a variety of physiological and pathological processes, including cell cycle regulation, mitochondrial biogenesis and function, glucose and lipid metabolism, insulin action, inflammatory responses, and energy homeostasis. In this review, we provided up-to-date and comprehensive knowledge about the roles of Sirtuins in adipose tissue remodeling, focusing on the fate of adipocytes, lipid mobilization, adipose tissue inflammation and fibrosis, and browning of adipose tissue, and we summarized the clinical trials of Sirtuin activators and inhibitors in treating metabolic diseases, which might shed light on new therapeutic strategies for obesity and its associated metabolic diseases.

## Introduction

Obesity has reached epidemic proportions globally in the past several decades, in both children and adults (1), which is a major contributor to the explosion of obesity-related metabolic diseases, including non-alcoholic fatty liver disease, cardiovascular diseases, and type 2 diabetes mellitus (2–4). Excessive energy is stored in adipose tissue (AT) in the form of triglycerides (TGs), causing obesity. Pathological AT expansion, which is accompanied by massive enlargement of existing adipocytes, the over-production of extracellular matrix (ECM), inadequate angiogenesis, elevated immune cell accumulation, pro-inflammatory responses, and ectopic lipid deposit, is termed AT remodeling (5). Massive expansion and remodeling of AT occur during obesity, and different AT depots exhibit various scenery (1). Under high-fat diet (HFD) feeding, visceral AT and subcutaneous AT expand through the enlargement of pre-existing adipocytes (hypertrophy) and the recruitment of newly generated adipocytes (hyperplasia), respectively; hyperplasia is metabolically healthy, whereas hypertrophy leads to metabolic complications (6). Adipose progenitors are a heterogeneous group of cells with diverse cell fates, contributing to white and beige adipogenesis, fibrosis, or maintenance of an immature cell phenotype with proliferation capacity. The factors shaping cell fate decisions of adipose progenitor cells determine the onset and development of obesity (7). Lipid mobilization, referring to fatty acid trafficking in (lipogenesis) and out (lipolysis) of the adipocytes, is a paramount process in regulating systemic energy metabolism. Hormone-sensitive lipase (HSL), adipose TG lipase (ATGL), and monoglyceride lipase (MGL) are considered the key rate-limiting enzymes responsible for lipolysis in adipocytes (8).

ECM components, such as fibronectin and collagen, provide mechanical support for hypertrophic adipocytes, while abnormal production and deposition of ECM cause the destruction of normal AT structure and impaired tissue flexibility in obese subjects (9). Adipocyte stress and death, and the formation of inflammatory foci occur when hypertrophic adipocytes lack proper ECM support (10). Evidence suggests that macrophages are the predominant leukocytes in AT, and the proportion of macrophages increases from approximately 5% in lean AT to more than 50% in obese AT (11). In addition, AT inflammation is accompanied by the shift from the alternatively activated macrophage (M2) phenotype to the classically activated macrophage (M1) phenotype in obese subjects (12). M1 macrophages secrete various pro-inflammatory cytokines, such as tumor necrosis factor-α (TNF-α) and interleukin-1β (IL-1β), and chemokines, such as monocyte chemoattractant protein-1 (MCP-1) and macrophage inflammatory protein-1α (MIP-1α). Infiltration and pro-inflammatory polarization of macrophages are determining factors of systemic inflammation and insulin resistance (IR) (13). Therefore, AT inflammation and abnormal ECM deposition play critical roles in obesity-induced metabolic disorders (14, 15). Unlike white AT, which stores energy, brown AT contains large amounts of mitochondria and dissipates lipids as heat by uncoupling protein 1 (UCP1) to maintain body temperature. Beige cells arise as multilocular adipocytes and have a highly inducible thermogenic capacity upon stimulation (16). Igniting white AT browning has become an attractive strategy for the treatment of obesity and its related metabolic disorders (17).

The silent information regulator 2 (Sir2) family of histone/protein deacetylases (Sirtuins) comprise homologs found across all kingdoms of life (18). There are seven Sirtuin homologs in humans, SIRT1–SIRT7. Sirtuins share significant sequence homology, contain conserved catalytic and nicotinamide adenine dinucleotide (NAD^+^)-binding domains, and regulate multiple cellular processes, including cell survival, senescence, and metabolic homeostasis (19). Sirtuins differ in their subcellular localization and substrate specificities. Mounting evidence indicated that Sirtuins are essential regulators of multiple processes in obesity-associated AT remodeling (20–23). Herein, we systematically summarized the roles of Sirtuins in regulating adipocyte fate, lipid mobilization, AT inflammation, AT fibrosis, and browning of AT (**Table 1**). The purpose of the current review is to provide insight into AT remodeling and inspire Sirtuins as therapeutic targets for obesity-associated metabolic disorders.

**Table 1:** Overview of the role of Sirtuins in adipose tissue remodeling.

	Adipocyte fate	Lipid mobilization	AT inflammation	AT fibrosis	AT browning
SIRT1	↓ Adipogenesis (24–26) by promoting CACUL1 binding to PPARγ-responsive site to repress PPARγ (24)	↑ Lipolysis by activating AMPK (20), and repressing PPARγ (27) and FOXO1-mediated expression of ATGL (28)	↓ Pro-inflammatory responses (29–43) by inhibiting NF-κB signaling pathway (29, 36–42), NLRP3 (34, 35), mTOR/S6K1 pathway (43), STAT3 (39), FOXO1-C/EBPα transcriptional complex (44), and PPARγ (45)	↓ ECM and macrophage infiltration (39)	↑ White AT browning (20, 27, 46–49) by deacetylating PPARγ (27, 48) and activating AMPK (20) and FGF21 (49)
	Not related to brown adipocyte differentiation (29)				
	SIRT1 deficiency suppresses adipogenesis by increasing the acetylation of NCOR1 during the early stage of mESCs to adipocyte differentiation (50)			SIRT1 deficiency ↑ ECM by suppressing the expression of leptin, adiponectin, and MMP3/13, and elevating the expression of Collagen 6A3 (51)	
	↑ Beige adipocyte differentiation of elderly AT-MSC *via* p53/p21 pathway (52)		↑ Anti-inflammatory responses (46, 53) by deacetylating the transcription factor NFATc1 (53)		
	↓ Lipid droplet number, lipid accumulation, and adipogenesis by preventing the proper induction of PPARγ2 and C/EBPα in visceral AT-derived stem cells (26)				SIRT1 deficiency ↑ brown AT degeneration by decreasing PGC-1α, UCP1, and CPT1b (54)
SIRT2	↓ Adipogenesis (55, 56) by deacetylating FOXO1 and promoting FOXO1 binding to PPARγ (56)	↑ Lipolysis (55, 57) by deacetylating PGC-1α (58)	Not available (N/A)	N/A	N/A
	↓ Lipid droplet number, lipid accumulation, and adipogenesis, by preventing the proper induction of PPARγ2 and C/EBPα in visceral AT-derived stem cells (26)				
SIRT3	↑ Brown adipocyte differentiation through PGC-1α (59)	↓ Lipid droplet size and accumulation (21, 60) by activating the AMPK-ULK1 pathway (60)	↓ Pro-inflammatory responses by inhibiting NLRP3 (61)	SIRT3 deficiency ↑ collagen VI (61)	SIRT3 deficiency ↑ brown AT whitening (21, 61, 62), suppresses UCP1 (61) and perilipin-1 (61, 62) and promotes collagen IV (61)
	SIRT3 deficiency promotes adipogenic differentiation by decreasing FOXO3a (63)				
	No effect on adipogenesis in 3T3-L1 cells (64)				
SIRT4	↑ Adipocyte differentiation by interacting coordinately with the transcription factors including C/EBPβ, E2F-1, and HOXA5 (65)	↑ Lipogenesis by repressing FAO via deacetylating MCD (66)	N/A	N/A	N/A
	↑ Adipogenesis by promoting branched-chain amino acid catabolism by MCC1 (64)				
SIRT5	↓ Adipocyte differentiation (67, 68), lipid synthesis, and lipid deposition by activating AMPK and repressing MAPK (68)	SIRT5 deficiency ↓ FAO (69) and ↑ lipolysis by stimulating ATGL (67)	N/A	N/A	SIRT5 deficiency ↓ AT browning (22, 69) by decreasing the expression of thermogenic genes including UCP1, CIDEA, COX7A1, CPT1b, and MCAD (21)
	↑ Adipogenesis modestly in 3T3-L1 cells (64)	No effect on lipolysis (70)			
SIRT6	↓ Adipocyte differentiation by activating AMPKα (71)	↑ Lipolysis *via* specific reduction of PPARγ signaling (72)	↓ Pro-inflammatory responses (23, 73, 74) by increasing the occupancy of c-Jun (73) and inhibiting NF-κB signaling pathway (74)	N/A	SIRT6 deficiency ↓ AT browning (75–78) by decreasing UCP1 (23, 76) and PGC-1α (76)
	↑ Adipogenesis (79) by inhibiting KIFC and enhancing CK2; SIRT6 deficiency leads to a severe adipogenesis defect and reduced expression of adipogenic markers, including PPARγ, C/EBPα, aP2, and adiponectin	SIRT6 deficiency ↓ lipolysis by suppressing the expression of ATGL by regulating FOXO1 acetylation (73)	↑ Anti-inflammatory responses (76, 80)		
SIRT7	↑ Adipogenesis by suppressing SIRT1 (81)	N/A	N/A	N/A	N/A

CACUL1, CDK2-associated cullin 1; PPARγ, peroxisome proliferator-activated receptor γ; FOXO, forkhead box O; ATGL, adipose triglyceride lipase; NLRP3, nucleotide-binding oligomerization domain, leucine-rich repeat and pyrin domain-containing 3; mTOR, mammalian target of rapamycin; ECM, extracellular matrix; AT, adipose tissue; NCOR1, nuclear receptor corepressor 1; mESCs, mouse embryonic stem cells; NF-κB, nuclear factor-κB; MMP3/13, matrix metalloproteinases 3/13; PGC-1α, proliferator-activated receptor-γ coactivator-1α; UCP1, uncoupling protein 1; CPT1b, carnitine palmitoyltransferase 1b; ULK1, unc-51-like kinase 1; E2F-1, E2F transcription factor-1; HOXA5, homeobox A5; MCC1, methylcrotonyl-CoA carboxylase 1; FAO, fatty acid oxidation; CIDEA, cell death-inducing DFFA-like effector a; COX7A1, cytochrome c oxidase subunit 7A1; MCAD, medium-chain acyl-coenzyme A dehydrogenase; KIFC, kinesin family member C; CK2, casein kinase 2; AMPK, adenosine 5′-monophosphate-activated protein kinase; MCD, deacetylating malonyl CoA decarboxylase; N/A, Not available.

## NAD^+^ in adipose tissue

As the rate-limiting co-substrate of Sirtuins, NAD^+^ is important to regulate the functions of Sirtuins and, consequently, AT remodeling. Emerging evidence has revealed that NAD^+^ biology in AT is associated with metabolic flexibility in mice and humans (82–84). NAD^+^ levels in white AT are decreased in obesity, which is consistent with the activity of Sirtuins (85, 86). Similarly, long-term HFD feeding reduced the content of NAD^+^ in epididymal white AT, inguinal white AT, and interscapular brown AT, while long-term calorie restriction (CR) showed the opposite effects; in addition, NAD^+^ content in both white and brown AT was negatively associated with the cholesterol and TNF-α levels in plasma but positively correlated with adiponectin level in plasma (87). Enhancing NAD^+^ synthesis in the salvage pathway plays a critical role in the differentiation of 3T3-L1 preadipocytes (88). Nicotinamide phosphoribosyltransferase (NAMPT) functions intracellularly to catalyze the rate-limiting step of the NAD^+^ salvage pathway, which is the main source of NAD^+^ in AT (83). Loss of NAMPT impairs metabolic pathways involved in inflammation (84) and decreases adrenergic-mediated lipolysis in white AT (82). Adipocyte-specific NAMPT deletion causes local AT inflammation, but not systemic inflammation (89). Nicotinamide riboside (NR) was recently identified as a NAD^+^ precursor; NR supplementation increases NAD^+^ levels and activates SIRT1 and SIRT3, ultimately enhancing oxidative metabolism in brown AT and protecting from metabolic abnormalities in HFD-fed mice (90). Additionally, NR supplementation prevents the development of inflammation and fibrosis in white AT of old, but not young, female HFD-induced-obesity mice (91). These findings demonstrated the importance of NAD^+^ biology in AT remodeling. Further studies are needed to clarify the source and functions of NAD^+^ in AT.

## Sirtuins in adipose tissue

SIRT1, existing primarily in the nucleus and partially in the cytoplasm, exhibits a deacetylase activity of histones and non-histone substrates, which manipulates multiple physiological processes in AT, including inflammatory responses, mitochondrial biogenesis, cellular senescence, and apoptosis/autophagy (92). SIRT2, originally identified as a tubulin deacetylase, is shuttled between the cytoplasm and the nucleus (93). SIRT2 participates in the regulation of adipocyte differentiation, gluconeogenesis, insulin action, and inflammatory responses by deacetylating various substrates (94). SIRT3, preferentially localized in mitochondria (95), possesses regulatory roles in multiple metabolic processes, including acetate metabolism and thermogenesis, by controlling mitochondrial biogenesis and function (96). SIRT3, rather than SIRT4 or SIRT5, is responsible for the overall protein deacetylation in mitochondria (97). SIRT4 and SIRT5 exist predominantly in mitochondria, whereas SIRT6 and SIRT7 are principally found in the nucleus.

The expression of Sirtuins in AT has been widely investigated, while the outcomes were controversial. The expression levels of SIRT2, SIRT4, and SIRT6 are comparable in white and brown AT, SIRT1 and SIRT7 maintain higher expression in white AT, and SIRT3 and SIRT5 are preferentially expressed in brown AT versus white AT (98). Compared with that of normal-weight individuals, the mRNA level of SIRT1 is lower and that of SIRT7 is higher in visceral and subcutaneous ATs from obese humans, while the expression of the other five Sirtuins is not related to body mass index (BMI) (99). In addition, the level of SIRT1 was found to be negatively correlated with BMI and AT macrophage infiltration in humans (100). The expression of SIRT1, SIRT3, and SIRT6 in subcutaneous AT was upregulated during weight loss (101). Consistently, the expression of SIRT1, SIRT3, and SIRT5 in subcutaneous AT is lower in the heavier co-twins of the BMI-discordant twin pairs (102). Another study showed that the mRNA expression of SIRT1, SIRT3, and SIRT7 is lower in subcutaneous AT of healthy obese subjects than that of lean subjects (86). Interestingly, overweight subjects showed lower SIRT3 and SIRT6 mRNA levels than normal-weight subjects; no differences in SIRT1 or SIRT6 levels were observed between obese and overweight subjects; the obesity group exhibited the highest expression, while overweight subjects showed the lowest expression of SIRT2 (103). The SIRT3 expression is relatively high at the initial stage of adipocyte differentiation and declines 4 days after hormonal stimulation in 3T3-L1 adipocytes (60). Inhibition of SIRT5 promoted the expression of SIRT6 in the differentiation process of bovine preadipocytes (71). Taken together, the expression profiles of Sirtuins and their functions in different physiologic contexts of AT vary, which in turn affect adipose tissue functions, and the change patterns are complicated and worthy of further exploration.

Diet and environmental stresses have been reported to target Sirtuins, as well as some small molecules. HFD feeding decreases the expression levels of SIRT1–4 and SIRT6 and increases the expression of SIRT5 in AT (104). Long-term CR increases the levels of SIRT1 (105, 106), SIRT2 (107), SIRT3 (98), and SIRT6 (108, 109) in AT but represses the level of SIRT4 (110). In addition, one report suggested no change of SIRT7 expression in white AT after short-term CR (111). Additionally, cold exposure upregulates the expression of SIRT1 (112), SIRT2 (55), SIRT3 (98), SIRT5 (22), and SIRT6 (75) in brown AT. It remains unclear how CR affects SIRT5 expression in AT and how cold exposure regulates SIRT4 and SIRT7 in AT.

## Sirtuins in manipulating adipocyte fate

During obesity, white AT expands excessively by increasing the size of pre-existing adipocytes or generating new adipocytes from precursor cells. The process of preadipocyte differentiation into mature adipocyte is termed adipogenesis. It is worth noting that adipocyte expansion *via* adipogenesis can offset the negative metabolic effects of obesity, and increasing reports are focused on exploring the mechanisms and regulators of this adaptation process (113). Among them, Sirtuins have attracted more and more attention.

### SIRT1–3 and SIRT7

Peroxisome proliferator-activated receptor γ (PPARγ) is one of the master regulators of adipocyte differentiation and is closely related to the development of obesity (24). Under fasting conditions, SIRT1 is activated to promote CDK2-associated cullin 1 (CACUL1) binding to PPARγ-responsive site without affecting CACUL1 expression, in turn repressing the transcriptional activity and adipogenic potential of PPARγ in 3T3-L1 cells (24). It has been reported that ECM in AT accelerates early adipogenesis, and SIRT1 mediates proadipogenic events triggered by ECM in subcutaneous AT (25). Conversely, SIRT7 promotes adipogenesis in mice by inhibiting the autocatalytic activation of SIRT1 (81). Strikingly, another report suggested that SIRT1 plays a positive role in the early stage of mouse embryonic stem cell (mESC) differentiation to adipocyte and exhibits a negative effect on the late stage of adipogenesis (50). SIRT1 deficiency increases the acetylation of nuclear receptor corepressor 1 (NCOR1) to inhibit adipogenesis; thus, SIRT1 plays a positive role during the early stage of mESCs to adipocyte differentiation (50).

In AT-derived mesenchymal stem cells (AT-MSCs), SIRT1 activated beige adipocyte differentiation in elderly AT-MSC *via* p53/p21 pathway, but not SIRT3 (52). On the contrary, another study indicated that the effects of SIRT1 on brown AT are not related to the differentiation process of brown adipocytes (29). The expression patterns of SIRT3 are correlated with brown adipocyte differentiation, and SIRT3 directly deacetylates PPAR coactivator-1α (PGC-1α) to promote the differentiation of fully thermogenic competent brown adipocytes (59). Depletion of SIRT3 decreases the protein level of forkhead box O 3a (FOXO3a) and subsequently impairs the ability of AT-derived human MSCs to undergo adipogenic differentiation, resulting in adipocyte dysfunction and IR (63).

It is worth noting that SIRT2 inhibits adipocyte differentiation. Apparently, the effects of SIRT1 and SIRT2 are similar in adipogenesis. Overexpression of SIRT1 and SIRT2 reduced lipid droplet number, attenuated lipid accumulation and adipocyte conversion, and prevented the proper induction of adipogenic markers, including PPARγ2 and CCAAT/enhancer-binding protein α (C/EBPα), in visceral AT-derived stem cells. Thus, the decreased expression of SIRT1 and SIRT2 promotes the differentiation capacity of visceral AT stem cells from obese humans, which is associated with fostering visceral AT expansion (26). SIRT2 directly deacetylates FOXO1 to affect FOXO1 acetylation/phosphorylation, increase its nuclear translocation, and suppresses adipogenesis (56). Moreover, SIRT2 promotes FOXO1 binding to PPARγ and subsequently inhibits its transcriptional activity to suppress adipogenesis (55).

### SIRT4–6

In bovine AT, SIRT4 interacts coordinately with the transcription factors, including E2F transcription factor-1 (E2F-1), C/EBPβ, and homeobox A5 (HOXA5), to promote adipocyte differentiation (65). SIRT4 promotes branched-chain amino acid catabolism through methylcrotonyl-CoA carboxylase 1 (MCC1), resulting in the greatest enhancement of adipogenesis, and SIRT5 only modestly promotes adipogenesis, but not SIRT3 (64).

A recent study reported that SIRT5 inhibition stimulates brown-like adipogenesis in 3T3-L1 preadipocytes (67). SIRT5 inhibits the differentiation of bovine preadipocytes and simultaneously inhibits lipid synthesis and lipid deposition in adipocytes by activating the adenosine 5′-monophosphate (AMP)-activated protein kinase (AMPK) and repressing the mitogen-activated protein kinase (MAPK) (68). Similarly, SIRT6 inhibits preadipocyte differentiation and synergizes with SIRT5 to reduce lipid deposition in preadipocytes through the activation of the AMPKα pathway (71). On the contrary, another study showed that SIRT6 promotes mitotic clonal expansion during adipogenesis by inhibiting the expression of kinesin family member C (KIFC) and enhancing casein kinase 2 (CK2) activity, while SIRT6 deficiency leads to a severe adipogenesis defect and reduced expression of adipogenic markers, including PPARγ, C/EBPα, adipocyte protein2 (aP2), and adiponectin (79).

**Figure 1:**
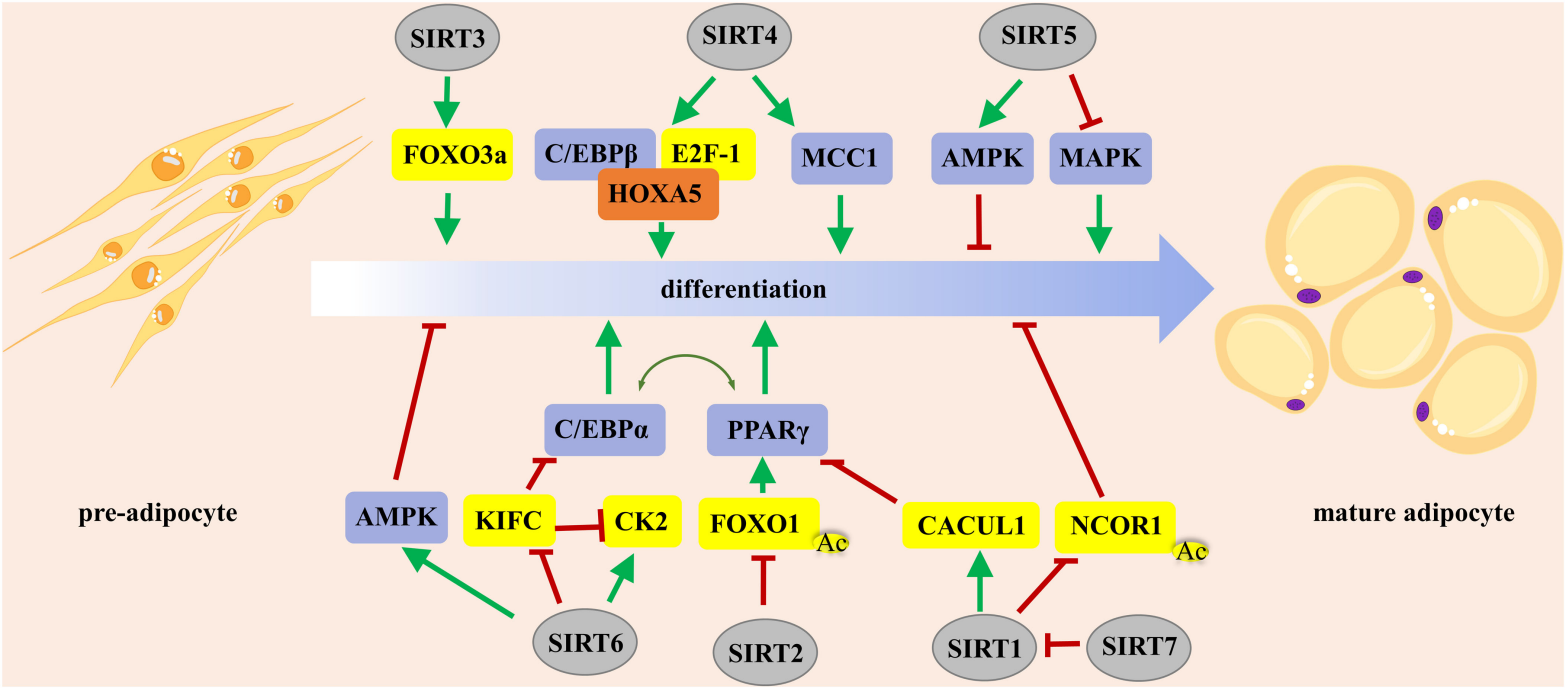
The roles of Sirtuins in regulating adipocyte differentiation. PPARγ and C/EBPα are the master regulators of preadipocyte commitment and terminal differentiation. FOXO plays an important role in the integration of hormone-activated signaling pathways with the complex transcriptional cascade that promotes adipocyte differentiation. PPARγ, peroxisome proliferator-activated receptor γ; FOXO, forkhead box O.

In summary, SIRT1 regulates adipogenesis either positively or negatively, which depends on the stage of adipocyte differentiation, whereas SIRT7 promotes adipogenesis by inhibiting the autocatalytic activation of SIRT1 (**Figure 1**). SIRT2 exerts an inhibitory effect on adipogenesis by deacetylating FOXO1. SIRT3 and SIRT4 promote adipogenesis, while SIRT5 and SIRT6 impair preadipocyte differentiation and lipid deposition. Manipulation of the activity of SIRT1–6 might be a promising strategy to control adipocyte fate.

## Sirtuins in regulating lipid mobilization

Obesity is characterized by the pathological expansion and dysfunction of AT. Lipogenesis is a process of the synthesis of fatty acids from non-lipid precursors. Lipolysis is a process of breaking down long-chain fatty acids to produce acetyl-CoA, which provides energetic needs for cells under physiological circumstances. Lipid mobilization, comprising lipogenesis and lipolysis, is critical for energy homeostasis. Sirtuins have received significant attention for their important roles in lipid mobilization in AT (114).

### SIRT1 and SIRT2

It was reported that upregulation of SIRT1 triggers lipolysis and loss of fat, including the hydrolysis of TGs and the release of free fatty acid (FFA), by repressing PPARγ (27). ATGL protein is considered the rate-limiting lipolytic enzyme since the rates of lipolysis are directly proportional to the levels of the ATGL protein. SIRT1 controls fat storage and mobilization, at least in part by regulating lipolysis in adipocytes *via* FOXO1-mediated expression of ATGL (28). Another study indicated that SIRT1 activates AMPK, which plays a crucial role in adipocyte lipolysis (20). Consistently, in white AT, SIRT1 knockdown not just fully recovered the resveratrol-elevated ATGL and positive regulatory domain containing 16 (PRDM16) protein but also reduced the resveratrol-elevated AMPK phosphorylation, suggesting that SIRT1 is the upstream factor of AMPK to control lipid metabolism (57).

SIRT2 increases lipolysis in mature adipocytes (55). SIRT2 mediates the increase in fatty acid oxidation (FAO) upon hypoxia-inducible factor-1α (HIF-1α) inactivation *via* PGC-1α, but not SIRT1 (58).

### SIRT3 and SIRT4

SIRT3 is able to prevent the loss of brown AT during obesity and metabolic disorders. Knockout of SIRT3 obviously promoted lipid droplet accumulation in brown AT (21). Interestingly, SIRT3 plays a minimal role in AT mitochondrial biology and systemic metabolism in adipocytes from SIRT3 knockout mice. Loss of SIRT3 in adipocytes showed no obvious change in metabolic responses to HFD feeding and aging (115). Overexpression of SIRT3 activated macroautophagy in mature adipocytes by activating the AMPK–unc-51-like kinase 1 (ULK1) pathway, which in turn resulted in smaller lipid droplet size and reduced lipid accumulation (60).

HFD feeding increases SIRT4 levels in mice (116), while in nutrient-replete conditions, SIRT4 is active to repress FAO by deacetylating malonyl CoA decarboxylase (MCD), an enzyme that produces acetyl-CoA from malonyl-CoA and stimulates lipogenesis (66).

### SIRT5 and SIRT6

SIRT5 deficiency impairs FAO, glutamate dehydrogenase (GDH) activity, and metabolic flexibility in brown adipocytes (69). In addition, a recent study reported that SIRT5 deficiency stimulates brown adipogenesis and ATGL function, which reduces intracellular lipid storage by promoting lipolysis and ultimately affects brown AT function (67). Strikingly, one study reported that SIRT5 deficiency did not cause any significant metabolic abnormalities under either chow or HFD conditions (70). Consistently, another study suggested there were no differences in the expression of genes related to fatty acid synthesis or transport, lipolysis, mitochondrial oxidative phosphorylation, or glucose transport in brown AT-specific SIRT5 knockout mice under standard housing conditions (22°C, normal chow diet) (69). Contradictory results might be due to different experimental subjects and conditions. Therefore, more studies are needed to confirm the effects of SIRT5 on lipid mobilization.

SIRT6 overexpression is associated with the downregulation of a selective group of PPAR-responsive genes and genes associated with lipid storage, including angiopoietin-like protein 4 (ANGPTL4), adipocyte fatty acid-binding protein (FABP4 or aP2), and diacylglycerol acyltransferase 1 (DGAT) (72). Fat-specific SIRT6 knockout sensitized mice to HFD-induced obesity, which was attributed to adipocyte hypertrophy instead of adipocyte hyperplasia by decreasing expression of ATGL. Furthermore, SIRT6 deficiency suppresses the expression of ATGL by regulating FOXO1 acetylation and subcellular localization, thereby inhibiting lipolytic activity (73).

**Figure 2:**
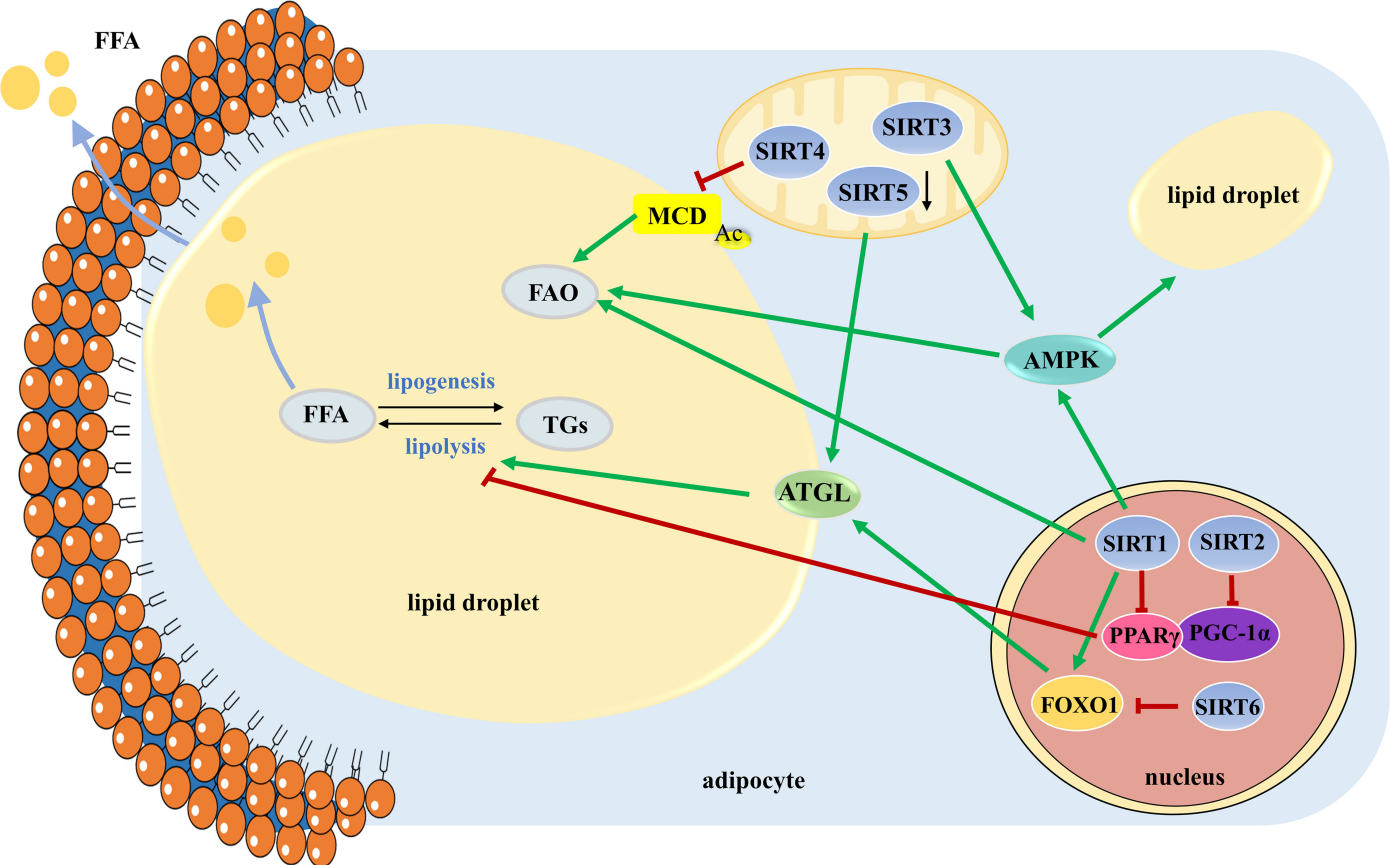
The roles of SIRT1-SIRT6 in manipulating lipid mobilization. Lipid mobilization is comprised of lipogenesis and lipolysis. Large lipid droplets are separated into small ones to initial lipolysis. TGs stored in lipid droplets are mobilized by the hydrolytic action of the three main lipases of the adipocyte to release FFAs, which are broken down to produce energy through FAO. TGs, triglycerides; FFAs, free fatty acids; FAO, fatty acid oxidation.

In conclusion, SIRT1 triggers lipolysis by repressing PPARγ, activating AMPK, and increasing the expression of ATGL. SIRT2 mediates the increase in FAO *via* PGC-1α. SIRT3 reduces lipid droplet size and lipid accumulation by activating the AMPK-ULK1 pathway. SIRT4 represses FAO by deacetylating MCD. The role of SIRT5 in lipid mobilization is controversial and needs further investigation. SIRT6 deficiency leads to decreased lipolytic activity by increasing phosphorylation and acetylation of FOXO1. In contrast, little is known about SIRT7 in lipid mobilization (**Figure 2**). Understanding the roles of Sirtuins in lipid mobilization would likely provide key insights into developing therapeutics against obesity and obesity-induced metabolic disease.

## Sirtuins in controlling adipose tissue inflammation

Elevating evidence suggests that the sequential course of inflammation is linked with immune responses, energy metabolism, and insulin sensitivity, which are regulated by Sirtuins (117). The roles of Sirtuins in acute/chronic inflammation have been summarized previously (117–119). AT is generally considered to be an active endocrine organ and takes a pivotal role in systemic energy homeostasis. AT inflammation, characterized by augmented infiltration and altered polarization of macrophages, results in IR and its associated metabolic diseases (15). In human stem cells from subcutaneous and visceral fat depots, the levels of Sirtuins 1-7 are involved in obesity-associated inflammation, as well as the interplay with PPARδ (120). Herein, the roles of Sirtuins in manipulating AT inflammation were summarized.

### SIRT1

SIRT1 has been reported to alleviate inflammation in a variety of tissues and cells (93). SIRT1 in AT plays a protective role against inflammation through multiple mechanisms. Macrophage infiltration and the gene expression of inflammatory cytokines were elevated in the heterozygous SIRT1 knockout mice fed with a moderate-fat diet (30). AT macrophages surround and ingest dying or dead adipocytes to form crown-like structures (CLSs). Myeloid SIRT1 deficiency promotes massive macrophage infiltration in AT and increases the number of CLSs (31, 32). Myeloid SIRT1 regulates pro-inflammatory cytokines and macrophage infiltration in AT from HFD mice (32). In addition, mice with AT-selective overexpression of human SIRT1 (H363Y), a dominant-negative mutant that inhibits endogenous SIRT1 activity, exhibited elevating inflammation (33). AT-specific-SIRT1 (H363Y) overexpressed mice exhibits hyperglycemia, dyslipidemia, and ectopic lipid deposition at a much younger age than their wild-type littermates (33). However, the pro-inflammatory effect of HFD is the triggering signal of SIRT1 cleavage.

In general, SIRT1 regulates both pro-inflammatory and anti-inflammatory cytokines. The pro-inflammatory signals that activate caspase-1 through the nucleotide-binding oligomerization domain, leucine-rich repeat and pyrin domain-containing 3 (NLRP3) inflammasome, such as TNF-α, cause the cracking of SIRT1. Indeed, AT-specific SIRT1 knockout mice obviously develop IR (34). In visceral AT, a negative correlation between the mRNA level of SIRT1 and IL-1β was observed (35). In addition, SIRT1 blocks the infiltration of macrophages and promotes the polarization toward anti-inflammatory M2 macrophages, which in turn ameliorates inflammation in AT (46); concomitantly, SIRT1 deacetylates the transcription factor NFATc1, thereby enhancing the binding of NFATc1 to the IL-4 gene promoter and finally modulating macrophage polarization (53).

Nuclear factor-κB (NF-κB) acts as a key regulator of inflammation to induce pro-inflammatory cytokines, which in turn increase adiposity and AT dysfunction (121). SIRT1 acts as a negative regulator of the inflammatory pathway and a positive regulator of insulin signaling in adipocytes by deacetylating NF-κB and inhibiting binding to the promoter of its target genes (36–40). Consistently, SIRT1 knockdown in white AT leads to NF-κB nuclear translocation by reducing histone H3 lysine 9 (H3K9) deacetylation (122). Interestingly, loss of SIRT1 leads to compensatory SIRT6 deacetylase activity on H3K9, demonstrating that SIRT1 and SIRT6 act on NF-κB through different mechanisms (40). Moderate SIRT1 overexpression ameliorates the effects of LPS on brown AT inflammation by the reduced acetylation of NF-κB, STAT3, and p38 MAPK (29, 39, 123). It is generally believed that the cluster of differentiation 40 (CD40)/CD40 ligand (CD40L) pathway is an integral part of the onset and maintenance of inflammatory reactions in obesity. SIRT1 modulates TNF-α-induced expression of CD40 partially *via* the NF-κB pathway in 3T3-L1 adipocytes (41). SIRT1 is a key upstream regulator in AT inflammation, by controlling the gain of pro-inflammatory transcription in response to inducers including fatty acids, hypoxia, and endoplasmic reticulum stress. The activation of SIRT1 by small molecules reduces the inflammatory response induced by FFA in macrophages and alleviates inflammation in white AT (38). SIRT1 suppresses NF-κB signaling pathway, which might provide another way to maintain AMPK activity against inflammatory challenges (42).

Phosphorylation of protein kinase B (Akt) activates the mammalian target of rapamycin (mTOR) signal in macrophages and then triggers inflammation and IR in obese mice (124). SIRT1 interacts with Akt2 and inhibits the mTOR/S6K1 pathway to attenuate AT inflammation in mice (43). Adiponectin, an adipocyte-derived relaxation factor with anti-inflammatory activity, promotes nitric oxide production in the endothelium. SIRT1 upregulates adiponectin mRNA expression in 3T3-L1 adipocytes *via* a FOXO1-C/EBPα transcriptional complex (44). SIRT1 improves the release of adiponectin from perivascular AT to fight against inflammatory insult (42).

Strikingly, adipocyte-specific deletion of SIRT1 exacerbates the detrimental effects of acute HFD feeding but shows protective effects in the context of chronic HFD exposure. Consistent with the more glucose tolerant and less insulin state of the adipocyte-specific SIRT1 knockout mice after chronic HFD, these mice possessed lower circulating levels of MCP-1 and TNF-α but increased levels of IL-10 and arginase in epididymal AT, which were mediated through hyperacetylation and dephosphorylation of PPARγ Ser273 along with reduced CDK5 activity. Additionally, increased p65 acetylation (NF-κB) was detected in these mice. Therefore, in the case of chronic HFD-induced obesity, inhibition of SIRT1 in adipocytes might result in improved metabolic functions (45). During the onset of obesity, SIRT1 deficiency in adipocytes (rather than myelocytes) accelerates peripheral IR by regulating macrophage infiltration and polarization, which has nothing to do with obesity (53).

Most studies have shown that SIRT1 plays a protective role in AT inflammation, but there are still very few studies with the opposite observation. The types of diet and the stage of obesity could affect the roles of SIRT1 in AT inflammation. Therefore, more studies are needed to further confirm the roles of SIRT1 in AT inflammation in the future.

### SIRT3 and SIRT6

The roles of other Sirtuins in AT inflammation have been seldom studied. SIRT3 mediates a metabolic switch in macrophages by deacetylating pyruvate dehydrogenase E1α (PDHA1) lysine, which in turn promotes NLRP3 inflammasome activation (61). Jun N-terminal kinase (JNK) in macrophages contributes to the accumulation of macrophages and plays a key role in the metabolic response to obesity, including IR (125). SIRT6 deficiency causes elevated inflammation in AT of mice by increasing the occupancy of c-Jun, downstream of JNK, on the gene promoters of IL-6 and MCP-1 (73). Another study suggested that SIRT6 regulates at least two stages of adipose inflammation: augmenting the migration potential of macrophages toward AT-derived chemoattractants and facilitating pro-inflammatory M1 polarization; SIRT6 deletion in macrophages promotes the activation of NF-κB and production of IL-6, resulting in STAT3 activation and the positive feedback circuits for NF-κB stimulation (74). Furthermore, adipocyte SIRT6 drives macrophage polarization toward M2 by increasing the production of the canonical type 2 cytokine IL-4 by adipocytes in a cell autonomous manner, which in turn attenuates pro-inflammatory responses in AT (80). Consistently, adipocyte SIRT6 decreases the M1 composition in white AT, and SIRT6 deficiency in adipocytes leads to an aggravating inflammatory reaction in white AT (23). Eosinophils secrete Th2 cytokine IL-4/IL-13 promoting M2 macrophage polarization. Myeloid-specific SIRT6 deficiency affects both eosinophils and M2 macrophage content in subcutaneous AT upon cold exposure, confirming a close link between eosinophils and M2 macrophages (76).

**Figure 3:**
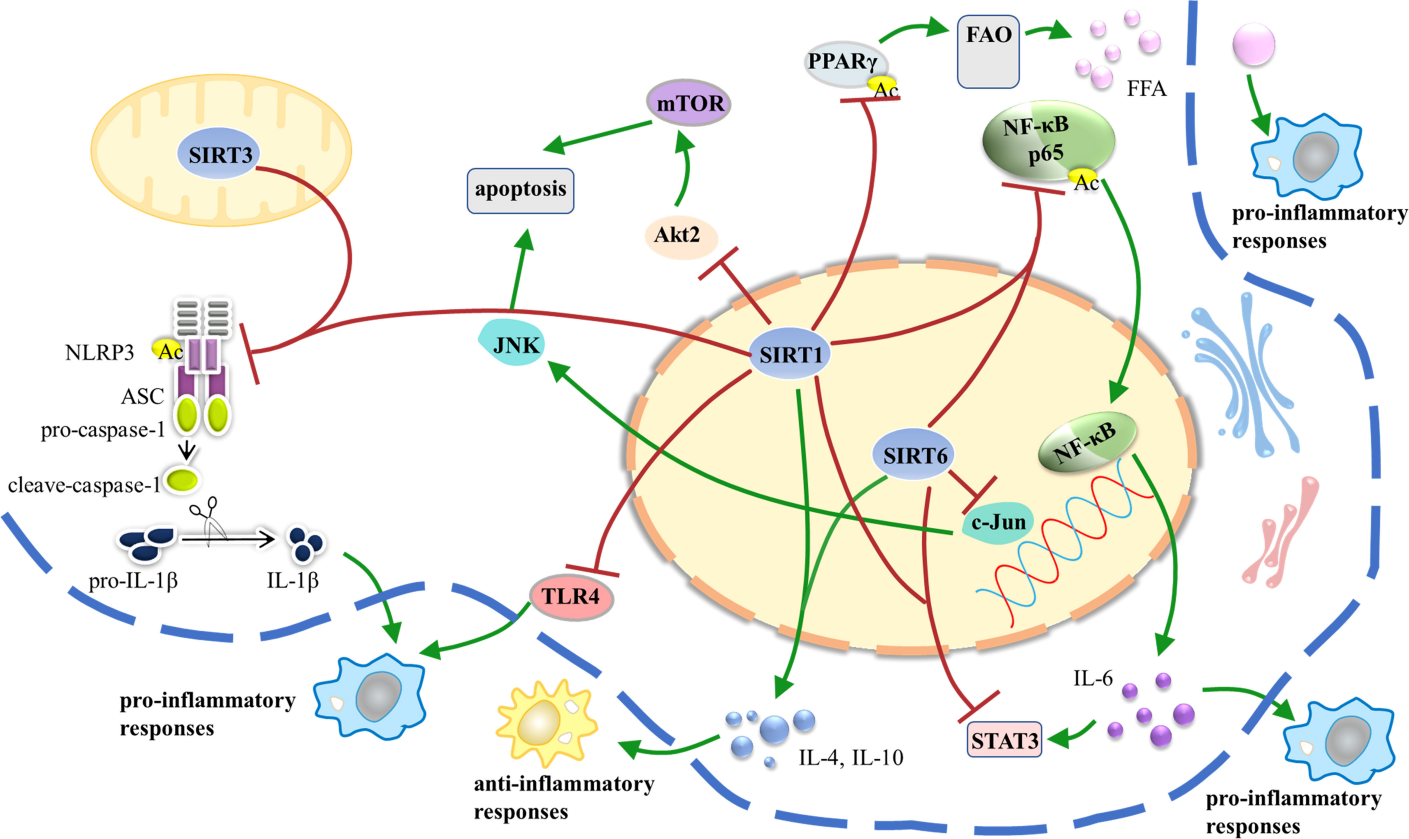
SIRT1, SIRT3, and SIRT6 participate in the regulation of AT inflammation *via* different pathways. AT inflammation is initiated and sustained over time by dysfunctional adipocytes that secrete inflammatory adipokines and by infiltration of bone marrow-derived monocytes that signal *via* production of cytokines and chemokines. White AT is the major source of obesity-related inflammation; in turn, AT inflammation leads to IR and metabolic dysfunction. AT, adipose tissue; IR, insulin resistance.

Taken together, SIRT1, SIRT3, and SIRT6 participate in the regulation of AT inflammation, while other Sirtuins remain poorly understood and need further researches (**Figure 3**).

## Sirtuins in manipulating adipose tissue fibrosis

ECM, a basic component of the specialized adipose niche, provides architectural elements and non-structural molecules that affect progenitor cells and regulate the expandability of AT (25). Fibrosis consists of excessive deposition of ECM, which eventually leads to organ failure and death in several chronic diseases (126). The important roles of Sirtuins in regulating AT fibrotic response have been gradually revealed.

### SIRT1

SIRT1 modulates fatty acid metabolism and lipid mobilization in adipocytes *via* modification of the extracellular environment. Small adipocytes, less ECM between adipocytes, and reduced macrophage infiltration in AT were observed in SIRT1 null animals (46). Consistently, SIRT1 deficiency suppresses the expression of leptin, adiponectin, and matrix metalloproteinases 3/13 (MMP3/13) and elevates the expression of the pro-fibrotic collagen (Collagen 6A3) in adipocytes. Pathway analysis revealed SIRT1-dependent key transcription factors, including PPARα, sterol regulatory element-binding transcription factor 1/2 (SREBF1/2), and PGC-1α (51). Moreover, SIRT1 overexpression downregulates the genes related to ECM remodeling (i.e., collagens, metalloproteases, and integrins) accompanied by a lower degree of inflammation-related fibrosis in visceral AT (39).

### SIRT3

Upon Angiotensin (Ang) II stimulation, adipocytes adjacent to the adventitia enlarge, while collagen IV deposition in perivascular AT increases. Myeloid SIRT3 deficiency resulted in severe loss of brown AT characteristics and increased expression of collagen VI, which ultimately aggravated Ang II-induced perivascular AT dysfunction (61).

SIRT1 participates in the regulation of AT fibrosis by controlling collagens and metalloproteases. The roles of other Sirtuins in AT fibrosis are seldom studied.

## Sirtuins in controlling adipose tissue browning

Browning of white AT is characterized by the induction of beige adipocytes, endowing brown AT-like characteristics onto white AT, and remodeling it to energy processing capacity in addition to energy storage capacity (54). AT browning plays a crucial role in energy metabolism, which could be a potential therapeutic strategy against obesity and metabolic syndrome.

### SIRT1

Pro-opiomelanocortin (POMC) neurons selectively regulate brown AT-like remodeling of perigonadal white AT, and SIRT1 deficiency affects the survival of POMC neurons (47). SIRT1 promotes AT browning in mice *via* the deacetylation of PPARγ on Lys268 and Lys293, which in turn recruits the transcriptional coactivator PRDM16 to PPARγ, resulting in selective induction of brown AT genes and repression of white AT genes (27, 48). It has been reported that SIRT1 regulates angiogenesis by modulating angiogenic factors (such as vascular endothelial growth factor, platelet-derived growth factor, and transforming growth factor-β), which in turn controls AT function (46). PGC-1α, highly expressed in brown AT, is a key factor of brown fat thermogenesis and white AT browning (20, 75). SIRT1 deficiency displays a lower thermogenic activity and a significant decrease of UCP1 and PGC-1α expression in brown AT from HFD-fed mice, which are accompanied by aggravated mitochondrial dysfunction. In line with this, oxidation genes were downregulated, including PPARα, peroxisomal acyl-coenzyme A oxidase (ACOX), and carnitine palmitoyltransferase 1b (CPT1b), and the mitochondria content was lower, ultimately leading to brown AT degeneration (54). Furthermore, SIRT1 is an endogenous activator of FGF21 in hepatocytes, which in turn systemically controls white AT browning and energy homeostasis (49). Another study reported that SIRT1 induces white AT browning following sleeve gastrectomy by activating AMPK (20).

### SIRT3

SIRT3 deletion aggravates brown-to-white adipocyte conversion induced by high salt *via* inhibiting mitochondrial biogenesis and perilipin-1 expression; however, restoring SIRT3 prevents high salt-induced brown AT to white AT conversion by improving mitochondrial respiration (62). Consistently, knockout of SIRT3 promotes the accumulation of lipid droplets in brown AT and blocks the inhibitory effect of capsaicin on HFD-induced brown AT whitening (21). Myeloid SIRT3 deficiency reduces UCP1 and perilipin-1 protein levels and promotes collagen IV deposition in brown AT, which in turn exacerbates perivascular AT remodeling and AT dysfunction (61).

### SIRT5

SIRT5 deficiency in mice resulted in less browning capacity in subcutaneous AT and a slight imbalance in energy and glucose homeostasis, which might be related to the inhibition of isocitrate dehydrogenase (IDH) activity and reduction of α-ketoglutarate concentration (22). In inguinal AT from SIRT5 knockout mice, the expression of thermogenic genes including UCP1, cell death-inducing DFFA-like effector a (CIDEA) and cytochrome *c* oxidase subunit 7A1 (COX7A1), and FAO genes, including CPT1b and medium-chain acyl-coenzyme A dehydrogenase (MCAD), was downregulated, while the expression of adipogenic genes (PPARγ and C/EBPα) was not changed (22). Another report suggested that mice lacking SIRT5 in brown AT showed difficulty converting fuel from FFA to glucose after overnight fasting. Strikingly, there was no difference in the expression of genes related to browning in brown AT-specific SIRT5 knockout mice (69).

### SIRT6

SIRT6 regulates AT browning in response to either cold exposure or β-adrenergic agonist (75). Similarly, SIRT6 deficiency in POMC neurons impairs the browning and lipolytic functions of AT in HFD-fed mice by modulating leptin signaling (77). Additionally, SIRT6 deficiency in mice resulted in impaired AT browning and reduced expression of UCP1, accompanied by downregulation of p38 MAPK/ATF2 signaling (23). SIRT6 deficiency leads to impairment of thermogenesis and browning by decreasing UCP1 and PGC-1α in subcutaneous white AT of myeloid SIRT6 knockout mice (76). SIRT6-deficient mice showed a significant increase in glucose uptake in brown AT, and HIF-1α is required to recruit SIRT6 to glycolytic gene promoters (78).

**Figure 4:**
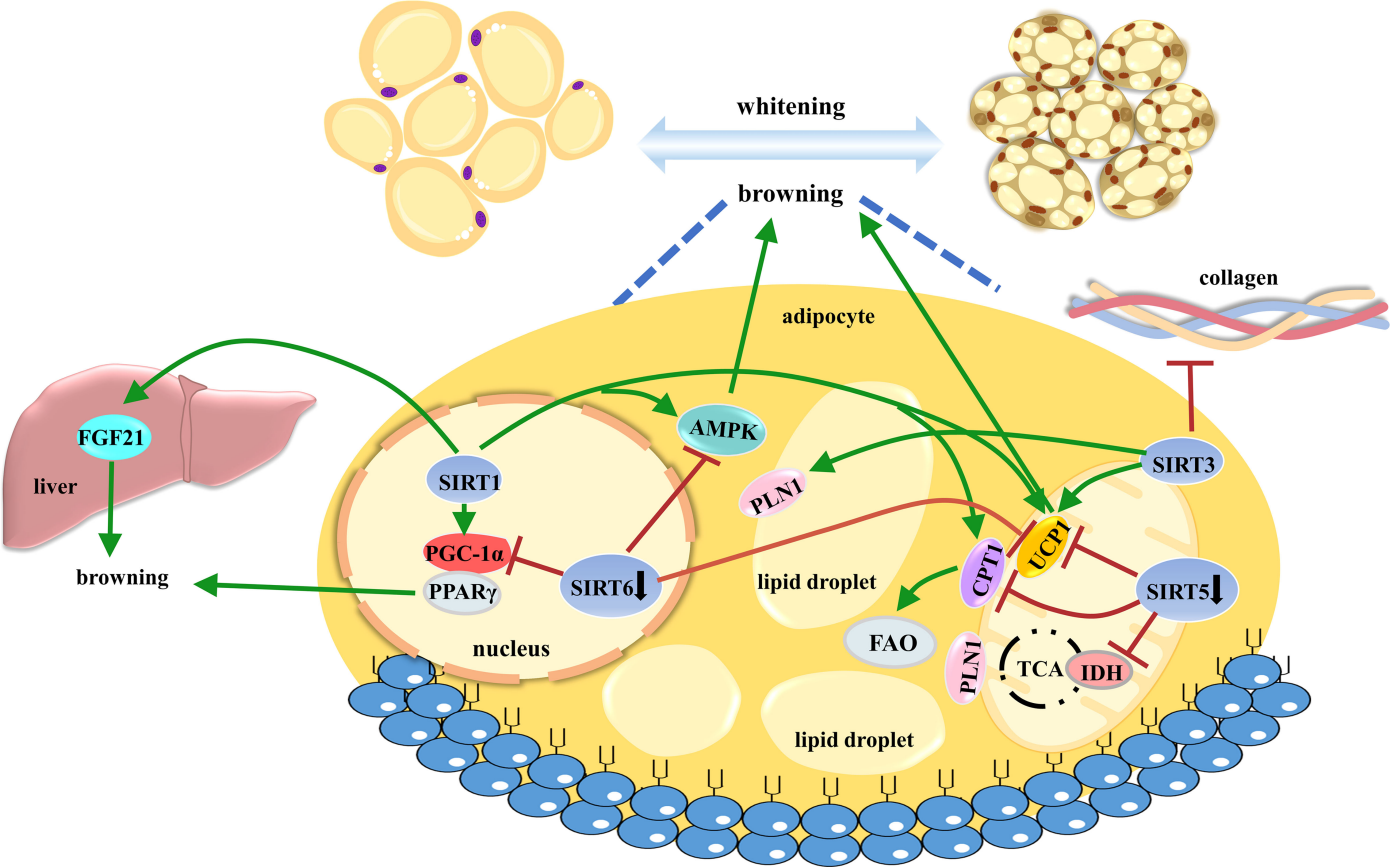
The roles of SIRT1, SIRT3, SIRT5, and SIRT6 in controlling AT browning. AT browning is a promising anti-obese strategy to enhance energy expenditure through heat production. Sirtuins widely participate in beige cell differentiation and function. AT, adipose tissue.

Taken together, emerging evidence has demonstrated that the Sirtuin family plays crucial roles in AT browning, comprehensively contributing to metabolic functions (**Figure 4**).

## Clinical progresses of Sirtuins

As Sirtuins are potential therapeutic targets for metabolic diseases, considerable efforts have been paid to develop specific Sirtuin activators and inhibitors in recent years. Unfortunately, there is still no drug approved for clinical use. Till now, one selective SIRT1 inhibitor, selisistat (also known as Ex-527 or SEN0014196), and several small-molecule SIRT1 activators have been evaluated in clinical trials (127). Data on Sirtuin activators and inhibitors are still limited, and their therapeutic efficacy remains under investigation. Considering Sirtuins are involved in multiple signaling pathways, the side effects of Sirtuin activators and inhibitors should be paid attention to in clinical trials. Here, we summarized the application of Sirtuin activators and inhibitors on obesity-related metabolic diseases.

### Inhibitor of Sirtuins

Selisistat, a selective SIRT1 inhibitor, was shown to be safe (128) and well tolerated in healthy volunteers and Huntington’s disease (HD) patients in short-term studies (129). Strikingly, a study showed that administration of selisistat over 14 days showed no pro-inflammatory effects (129), although some pre-clinical studies suggested its pro-inflammatory effect.

### Activators of Sirtuins

Resveratrol was identified as the most potent SIRT1 activator (130). One study showed that 150 mg/day trans-resveratrol (99.9%) supplementation in obese subjects for 30 days decreased the expression levels of inflammation-related genes, plasma levels of several inflammatory markers, and leukocyte numbers, and reduced AT lipolysis (131). However, resveratrol supplementation (75 mg/day) in non-obese, postmenopausal women with normal glucose tolerance did not change body composition, resting metabolic rate, plasma lipids, or inflammatory markers (132). Longevinex is a modified form of resveratrol; it had no effect on insulin sensitivity or the inflammation markers (IL-6) in 34 patients diagnosed with metabolic syndrome (133).

Because resveratrol suffered from low bioavailability and potency, as well as low target specificity, synthetic Sirtuin activators are emerging, such as SRT501, SRT2104, SRT2379, and SRT3025 (127). SRT501 has entered phase III clinical trials for the treatment of type 2 diabetes, and the pharmacokinetics and safety study of SRT2379 evaluated in healthy male volunteers have been completed (130). The promising clinical data on SRT2104 revealed that small-molecule SIRT1 activators with good pharmacokinetics and tolerability profiles could provide important new therapeutic paradigms and be developed as candidates to treat inflammatory diseases (134, 135). There is still no Sirtuin activator or inhibitor approved for clinical use; further structural modifications, pharmacological evaluations, and clinical trials are needed.

## Perspectives

Understanding the roles of Sirtuins in AT remodeling could help to untangle the comprehensive regulatory circuits of obesity. Each member of the Sirtuin family participates in the regulation of adipogenesis, lipid mobilization, AT inflammation, AT fibrosis, and AT browning through multiple pathways. Among them, SIRT1 has been widely investigated with multiple functions in AT remodeling. Specifically, SIRT1 suppresses adipogenesis by modifying the activity of PPARγ in preadipocyte and white AT; triggers lipolysis by repressing PPARγ and activating the AMPK pathway; exerts anti-inflammatory effect by repressing NF-κB, NLRP3, and mTOR pathways; and regulates ECM deposition and AT fibrosis. SIRT3 not only promotes brown adipocyte differentiation but also stimulates thermogenesis. SIRT3 reduces lipid droplet size and lipid accumulation by activating the AMPK pathway and mediates NLRP3 inflammasome activation to exhibit an anti-inflammatory effect. SIRT6 suppresses preadipocyte differentiation and lipid deposition through the activation of the AMPK pathway. However, the roles of other Sirtuins in AT remain poorly understood. Adipocyte-, leukocyte-, or myeloid-specific knockout or knockin animal models are powerful tools to investigate the physiological function of Sirtuin deacetylases and their possible cross-regulation in AT remodeling. It should be noted that the pathway of Sirtuins and the interaction between Sirtuins vary greatly in different models, which need further exploration. Since the functions of Sirtuins are related to the stage of adipocyte differentiation, cell types (preadipocytes, white adipocytes, brown adipocytes, or beige adipocyte), and tissues (white or brown AT), the studies about Sirtuins on AT remodeling should be more comprehensive. Sirtuins mainly play protective roles in AT remodeling, while the effects of Sirtuin inhibitors and activators on obesity and obesity-related metabolic diseases remain elusive.

In summary, AT remodeling is a series of physiological and pathological responses of AT under the challenge of positive energy. By regulating AT remodeling, Sirtuin deacetylases could revolutionize obesity and its related complications. This review may contribute to a better understanding of AT remodeling in obesity and the possible development of Sirtuins as new therapeutic targets.

## Author contributions

All authors contributed to the study’s conception and design. Material preparation, data collection, and analysis were performed by JC and RL. The first draft of the manuscript was written by JC, and all authors reviewed subsequent drafts and have approved the final version for submission. All authors contributed to the article and approved the submitted version.

## Funding

Financial support by the Open Research Fund of Chengdu University of Traditional Chinese Medicine Key Laboratory of Systematic Research of Distinctive Chinese Medicine Resources in Southwest China (2022ZYXK2011007), National Natural Science Foundation of China (81872754, 82073715), the Research Fund of University of Macau (MYRG2020-00091-ICMS), Internal Research Grant of the State Key Laboratory of Quality Research in Chinese Medicine, University of Macau (QRCM-IRG2022-014), and the Science and Technology Development Fund, Macao SAR (FDCT 0064/2021/AGJ), is gratefully acknowledged.

## Conflict of interest

The authors declare that the research was conducted in the absence of any commercial or financial relationships that could be construed as a potential conflict of interest.

## Publisher’s note

All claims expressed in this article are solely those of the authors and do not necessarily represent those of their affiliated organizations, or those of the publisher, the editors and the reviewers. Any product that may be evaluated in this article, or claim that may be made by its manufacturer, is not guaranteed or endorsed by the publisher.
